# IFN-γ+ NK cells as a potential predictor of pregnancy loss in unexplained recurrent pregnancy loss

**DOI:** 10.7150/ijms.121925

**Published:** 2026-05-29

**Authors:** Niwei Yan, Pingyin Lee, Huiying Jie, Lingli Long, Yu Fu, Canquan Zhou, Yuan Yuan

**Affiliations:** 1 Center for Reproductive Medicine, The First Affiliated Hospital, Sun Yat-sen University, Guangzhou 510080, China.; 2 Guangdong Provincial Key Laboratory of Reproductive Medicine, Guangzhou 510080, China.; 3 LKS Faculty of Medicine, The University of Hong Kong, Pokfulam, Hong Kong.; 4 Clinical Trials Unit, The First Affiliated Hospital, Sun Yat-sen University, Guangzhou 510080, China.; 5 Center for Reproductive Medicine, Hainan Women and Children's Medical Center, Haikou, China.

**Keywords:** embryo implantation, interferon-gamma, Natural Killer Cells, recurrent early pregnancy loss

## Abstract

**Background:**

Natural Killer (NK) cells are the largest population of lymphocytes in the endometrium during early pregnancy and play a key regulatory role in trophoblast cell invasion and embryo implantation at the maternal-fetal interface. Previous studies on the roles of NK cells with distinct phenotypes in pregnancy loss were based on NK cells derived from the peripheral blood or decidua, and it is difficult to determine whether the reported changes in decidual NK cells are causes or consequences of pregnancy loss.

**Objective:**

We aimed at exploring the functional NK cells during the implantation window and their associations with pregnancy outcome.

**Methods:**

Using flow cytometry, we assessed the expression of functional markers including Interferon-gamma (IFN-γ) and CD107a in NK cells from unexplained recurrent pregnancy loss** (**uRPL**)** patients following coculture with HTR-8/SVneo trophoblast cells during the implantation window, then the associations of functional NK cells with early pregnancy outcomes (≤12 weeks) were explored.

**Result:**

In this exploratory cohort, we found that an increase in the frequency of CD3^-^CD56^+^IFN-γ^+^ uNK cells was associated with the recurrence of pregnancy loss in uRPL.

**Conclusion:**

Elevated CD3⁻CD56⁺IFN-γ⁺ uNK cells during the implantation window were associated with early pregnancy loss (≤12 weeks) in women with uRPL, suggesting a potential biomarker that requires validation in larger studies.

## Introduction

The concept of recurrent pregnancy loss (RPL), which was first introduced as three or more consecutive early pregnancy losses, has been changed as the distribution of associated factors in couples with two versus three or more pregnancy losses is equal 1. Therefore, most experts throughout the world now seriously consider two consecutive pregnancy losses as suitable for a diagnosis of RPL 2, 3.

Most pregnancy losses occur in the first trimester, and factors considered to be associated with RPL include uterine anomaly, antiphospholipid antibody syndrome (APS), endocrine abnormality, thrombophilia, abnormal parental chromosome, and abnormal embryonic karyotype 4, each of which can occur alone or in combination. In China, most primary health care institutions do not incorporate chorionic villus chromosomal testing for pregnancy loss into routine clinical practice because this method is limited by its time-consuming nature and maternal contamination, which interferes with the accuracy of the results. Nevertheless, nearly 30% of patients can be diagnosed with RPL after undergoing a series of routine etiologic examinations, and up to 75% of them are diagnosed with unexplained recurrent pregnancy loss (uRPL) 4.

The majority of scholars are supportive of an immunological basis for uRPL, but clinical evidence fails to support proven therapies. A gray market has flourished, with immunotherapies such as lymphocyte immunotherapy, intravenous immunoglobulin (IVIG), and fat emulsion injection offered to treat uRPL, although the guidelines have not recommended any therapeutic interventions to increase the live birth rate for couples with RPL with suspicion of an immunological background. Thus, it is of great significance to explore the predictors of uRPL and develop a highly targeted, efficient and less expensive therapeutic approach.

uRPL is speculated to be related to abnormal immunity at the maternal-fetal interface. Uterine natural killer (uNK) cells, which originate mainly from the migration and differentiation of peripheral blood NK(pbNK) cells 5, 6, reach a peak (70-80%) in the mid-luteal phase of embryo implantation and constitute the largest population of lymphocytes at the maternal-fetal interface in early pregnancy 7, 8. In the endometrium, uNK cells regulate the immune balance and secrete a large number of cytokines, which are dominant cytokines during pregnancy 9, 10. Most studies have confirmed that the number of uNK cells is associated with RPL 11, 12, yet a few studies have reported that there was no difference in the number of uNK cells between patients with RPL and normal reproductive-aged women 13, 14. However, immunohistochemical findings have consistently confirmed that the uNK cell count is higher in the luteal phase in women with RPL 12, 15, 16.

uNK cells were regarded as equivalent to pbNK cells because of their cytotoxic function. However, CD3^-^CD56^dim^CD16^+^ cells that constitute 20%-30% of uNK cell population are the majority of the pbNK cell population, and they have cytotoxic functions; conversely, CD3^-^CD56^bright^CD16^-^ cells constitute 70%-80% of the uNK cell population^17^, ^18^, and they primarily perform secretory and regulatory functions and can produce a range of cytokines, including vascular growth factors 19. Consequently, uNK cells are not considered to be associated with cytotoxic responses against embryos. The current knowledge on uNK cells in the endometrium during the menstrual cycle, especially in the decidua, suggests that these cells are key regulators during the first trimester, playing fundamental roles in vascular remodeling and trophoblast invasion 19. Therefore, functional changes in uNK cells could compromise the success of embryo implantation.

The cytokine environment at the maternal-fetal interface critically regulates uNK cell function and trophoblast development. A complex network of cytokines modulates trophoblast invasion and immune tolerance. IL-3, in particular, has been demonstrated to stimulate trophoblast proliferation and invasiveness, and decreased IL-3 production has been observed in conditions associated with pregnancy loss, such as antiphospholipid syndrome 20. This underscores the importance of the cytokine in supporting successful implantation. However, the functional status of NK cells themselves—particularly their production of effector cytokines like IFN-γ and their degranulation capacity—may more directly reflect the net outcome of these complex cytokine interactions at the maternal-fetal interface.

uNK cells at the maternal-fetal interface must interact with ligands on chorionic trophoblast cells through uNK cell-expressed receptors to play fundamental roles in embryo adhesion, implantation, and invasion Supplementary Table 1. Decidual NK (dNK) cells respond to invading EVTs (Extravillous Trophoblast) via expressed receptors, and the binding of dNK cell-expressed receptors with HLA ligands expressed by EVTs regulates trophoblast invasion and pregnancy outcome via the production of a large number of cytokines and angiogenic factors, resulting in embryonic immune escape, early chorion development and placenta formation 21-23.

Current knowledge on NK cell function is largely derived from peripheral blood or decidual tissues obtained after pregnancy loss, while NK cells directly obtained from the endometrium during the embryo implantation window have not yet been explored. It is difficult to determine whether changes in dNK cells are causes or results of pregnancy loss and whether the aforementioned findings for pbNK and dNK cells are applicable in the earliest stage of pregnancy when NK cells interact with embryonic trophoblast cells. To model the functional crosstalk between endometrial NK cells and trophoblasts during early pregnancy, we employed an *in vitro* co-culture system combining peripheral blood/endometrial lymphocytes (isolated during the implantation window) with the HTR-8/SVneo trophoblast cell line. This system was designed to model key initial interactions between endometrial lymphocytes and trophoblast cells during the implantation window, as HTR-8/SVneo cells express HLA-I molecules critical for NK cell recognition 24, 25 and mimic early gestational trophoblasts 26, while endometrial lymphocytes reflect the *in vivo* NK cell population. This study allows direct investigation of NK-trophoblast interactions underlying uRPL.

## Materials and Methods

### Subjects

**Figure 1:**
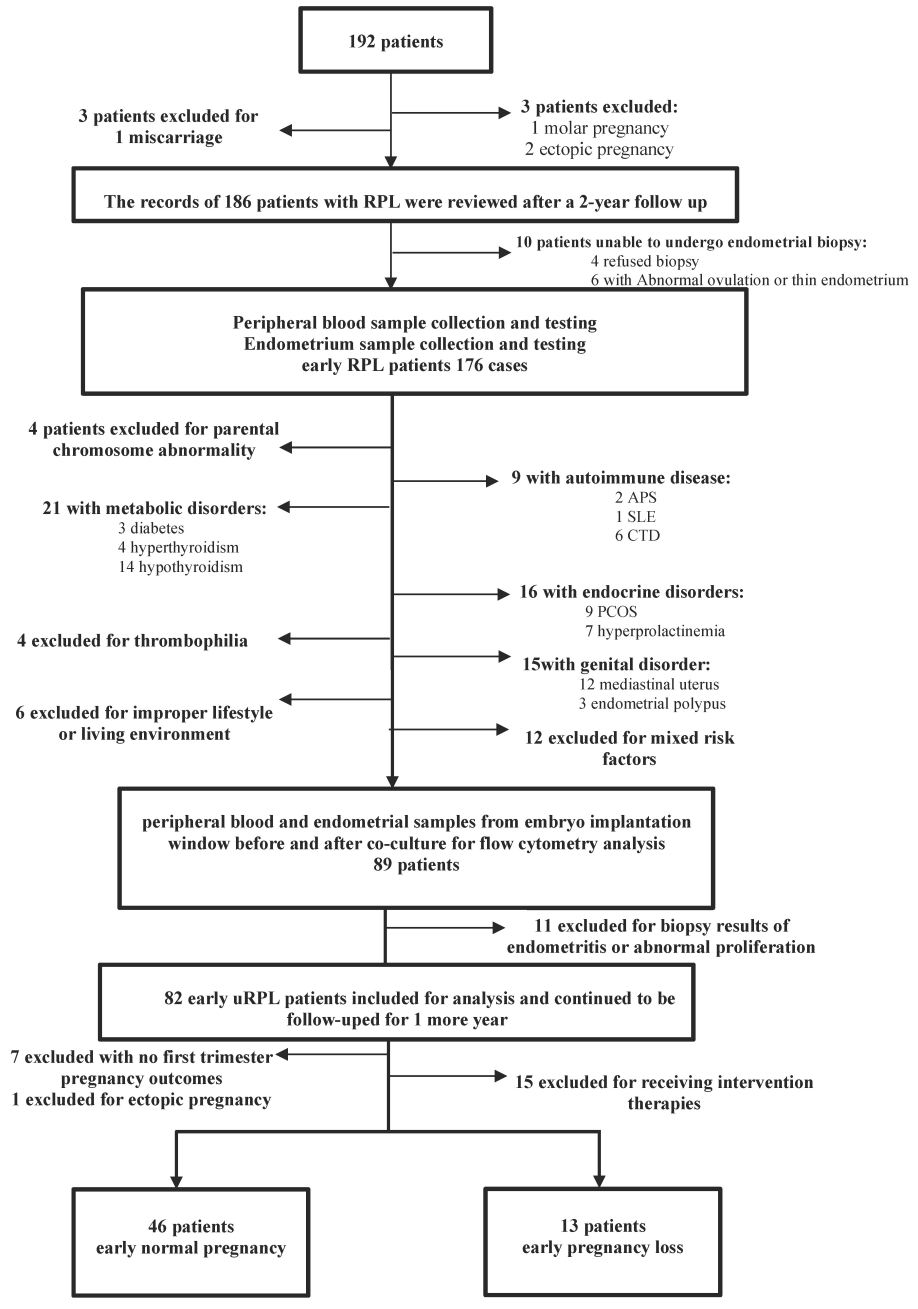
Flow chart of study participants.

The records of 192 patients who visited the specialist clinic at the First Affiliated Hospital of Sun Yat-sen University for RPL between November 2019 and August 2022 were carefully reviewed after a 2-year follow-up period. All of these patients finished routine etiologic examinations, including parental karyotyping, screening for uterine abnormalities, three-dimensional ultrasonography, immunological tests and evaluations for hereditary thrombophilia, antiphospholipid antibodies (lupus anticoagulant and anticardiolipin antibodies), thyroid function and antibodies, and endocrine and metabolic disorders. Six of the patients did not meet the diagnostic criteria for RPL, while 186 women were diagnosed with early RPL (within 12 weeks). Finally, a detailed flowchart of patient selection and follow-up is presented in Figure 1, according to the pregnancy outcome results (≤ 12 weeks), 59 patients without any intervention were divided into two groups, with 46 women in the early normal pregnancy (ENP) group and 13 women in the early pregnancy loss (EPL) group.

### Sample collection and lymphocyte isolation

All enrolled patients had a regular menstrual cycle and began to monitor ovulation regularly using ovulation kits combined with vaginal ultrasound at approximately 10 days after the beginning of menstruation. Follicle size was determined by ultrasound and recorded. When the test line appeared to be as dark as the control line (C = T), peripheral blood was collected to test the levels of hormones, including follicle-stimulating hormone (FSH), estradiol (E_2_), luteinizing hormone (LH) and progesterone (P). Endometrial biopsy was performed on the 7^th^ day after the LH surge (LH+7), and ovulation was confirmed by ultrasound.

Peripheral blood samples were collected on LH+7 to determine the NK cell count and mid-luteal E2 and P levels, while 3 ml of peripheral blood was retained for coculture with trophoblast cells and flow cytometry analysis. Endometrial samples were collected immediately after peripheral blood collection. Informed consent was obtained from each patient before endometrial biopsy. A small fraction of endometrial tissue was used for CD138 testing to rule out the possibility of endometritis, while most was retained for coculture with trophoblast cells and flow cytometry analysis. Three milliliters of fresh peripheral blood were collected in a 3-ml BD Vacutainer tube before endometrial biopsy, endometrial tissues were placed in 15-ml sterile centrifuge tubes after the procedure, and both were taken to the laboratory ultraclean table within 2 hours. Peripheral blood lymphocyte and endometrial blood lymphocyte isolation were further performed to obtain single-cell suspensions of lymphocytes Figure S1A-C, Figure S1F-H).

### Lymphocyte-HTR-8/SVneo human chorionic villus trophoblast cell coculture

The HTR-8/SVneo cell line was purchased from Procell Life Science & Technology Co., Ltd. (Wuhan, China). Cells were cultured in a T25 cm^2^ flask at 37 °C and 5% CO_2_ in a humidified atmosphere, and the medium was replaced with conditioned medium (DMEM+10% FCS+1% penicillin/streptomycin+1% diluent estradiol/progesterone) after testing negative for mycoplasma by polymerase chain reaction (PCR). HTR-8/SVneo trophoblast cells were subcultured using 0.25% trypsin-EDTA and seeded (1.0 × 10^5^ cells per well) in a 6-well plate (Corning, USA). A schematic diagram of this co-culture system is provided (Figure S2.

### Flow cytometry analysis

Single-cell suspensions of peripheral blood or endometrial lymphocytes tested negative for bacterial pathogens by PCR before coculture, and the culture medium of HTR-8/SVneo trophoblast cells was discarded. Then, an endometrial lymphocyte suspension (1.0 × 10^5^ lymphocytes per well) was added to the surface of the HTR-8/SVneo trophoblast cells (1.0 × 10^5^ cells per well) for coculture for 6 hours at 37 °C and 5% CO_2_ in a humidified atmosphere Figure S1D, Figure S1I) and prepared for multiparameter flow cytometry. For intracellular IFN-γ staining, protein transport inhibitor (Brefeldin A and Monensin) was added during the final 4 hours of the 6-hour co-culture period.

NK cells were identified as CD3ˉCD56+ lymphocytes. Subsets were assessed using the following fluorochrome-conjugated antibodies: anti-CD107a (clone H4A3, BD), anti-IFN-γ (clone 4S.B3, BD), and other subset markers (detailed in Supplementary Table 2. Data were acquired on a BD LSRFortessa 20x instrument and analyzed using FlowJo v10. For functional characterization, degranulation was assessed by CD107a surface expression on CD3ˉCD56⁺ gated cells, and IFN-γ production was measured by intracellular staining in CD3ˉCD56⁺ gated populations.

**Figure 2:**
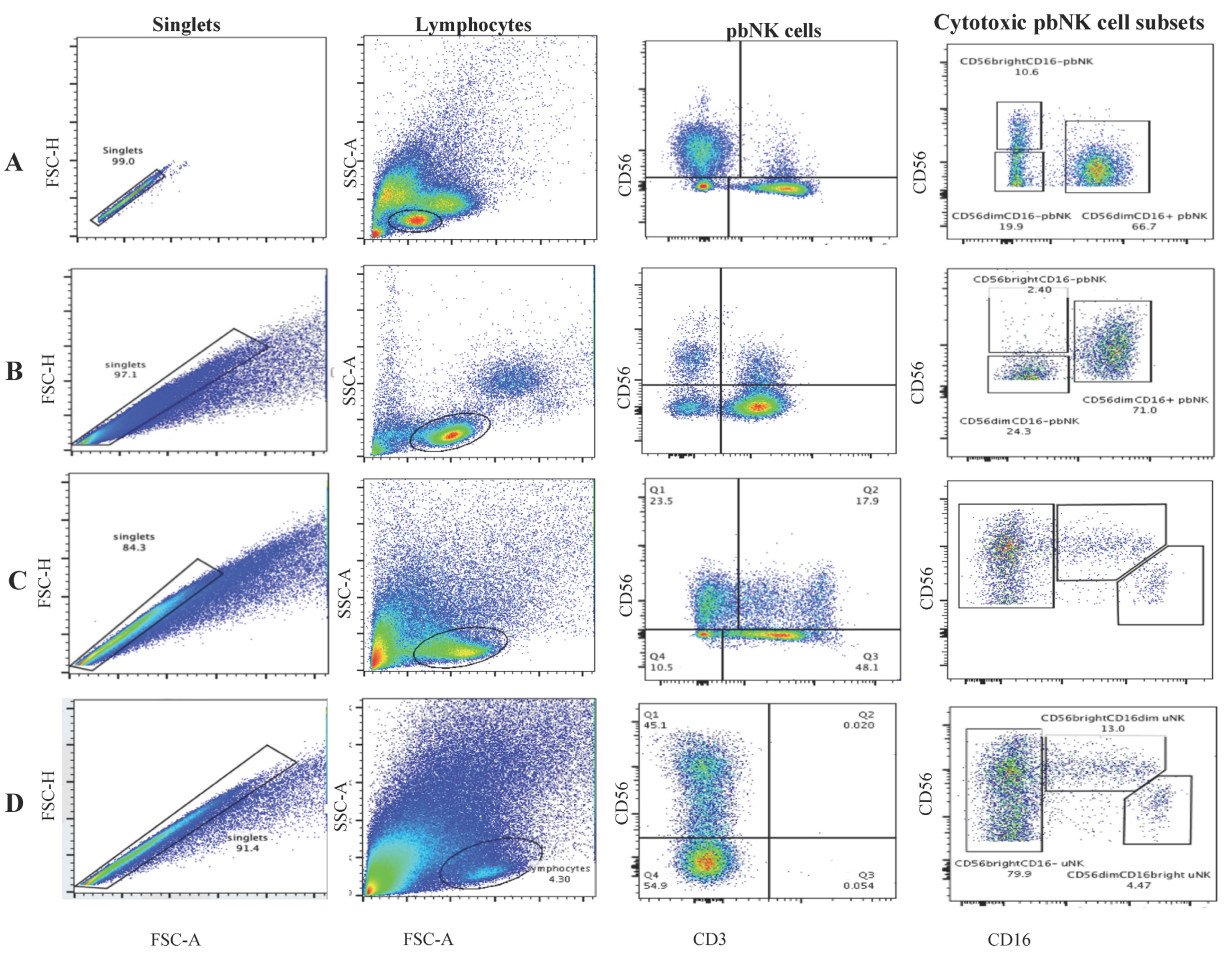
** Representative flow cytometry gating strategy for NK cell subsets.** (A-B) Peripheral blood NK (pbNK) cells were gated as CD3⁻CD56⁺ lymphocytes and further classified into CD56dimCD16⁺ and CD56brightCD16⁻ subsets in (A) the ENP group and (B) the EPL group. (C-D) Uterine NK (uNK) cells were gated as CD3⁻CD56⁺ lymphocytes and classified into CD56^bright^CD16⁻, CD56^bright^CD16^dim^, and CD56^dim^CD16^bright^ subsets in (C) the ENP group and (D) the EPL group.

The gating strategy for NK cell subsets is detailed in Figure 2. Briefly, after doublet exclusion, lymphocytes were gated based on FSC/SSC characteristics. NK cells were defined as CD3⁻CD56⁺ cells. From this population, CD56^bright^ and CD56^dim^ subsets were further delineated based on CD16 expression. For functional assays, baseline (unstimulated) levels of CD107a and IFN-γ were established using lymphocytes cultured alone. Appropriate concentrations of antibodies were added to the cells (5×10^5^ cells/tube) in 100 μL of flow cytometry staining buffer and incubated for 30 minutes at 4 °C in the dark. At least 50,000 lymphocyte-gated cells were obtained and analyzed for CD3-CD56+ cells. The normal range for the CD56+ pbNK cell frequency was less than 18% according to our laboratory standard. The flowchart of the experiment was shown in Figure S2.

### Statistical methods

Data are expressed as the mean ± standard deviation (mean ± SD), and the Shapiro‒Wilk method was used to examine the normality of data distributions. A t test for normally distributed data and the Mann‒Whitney U test for data with a nonnormal distribution were applied to compare study groups. Count data are expressed as a rate (%), and the presence of intergroup differences was assessed using the chi-square test. Data were analyzed using Statistical Package for Social Science (SPSS) version, and graphical analysis was performed using Prism GraphPad 9. Variables were selected for inclusion in the multivariable regression model based on two criteria: 1 established clinical relevance to RPL (maternal age, BMI, and history of previous pregnancy loss), and 2 a univariate association with pregnancy outcome at a threshold of p < 0.10 (frequencies of CD3⁻CD56⁺IFN-γ⁺ uNK and CD3⁻CD56⁺CD107a⁺ uNK cells). The final model was constructed using the backward elimination method. The results were considered statistically significant when the P value was less than 0.05. The relevant influencing factors of pregnancy outcomes were determined via multivariable regression analysis with the backward method. The results were considered to be statistically significant when the P value was less than 0.05 (*P < 0.05, **P < 0.01, **P < 0.001, and ***P < 0.0001).

### Sample size calculation

The sample size was determined based on preliminary data showing a 0.09 difference (SD = 0.08) in NK cell levels between groups. With α = 0.05 (two-sided), 80% power, and 3:1 allocation ratio, we required 25 successful-pregnancy and 9 pregnancy-loss cases. Accounting for 10% attrition, we aimed to enroll 28 and 10 cases respectively (total N = 38). The study ultimately included 46 successful-pregnancy and 13 pregnancy-loss cases, exceeding the minimum requirement.

### Ethics statement

Research Protocol was approved by Independent Ethics Committee for Clinical Research and Animal Trials of the First Affiliated Hospital of Sun Yat-sen University on June 22, 2016 under approval number IIT-2016-114.

## Results

The records of 192 patients were reviewed and 59 patients without any intervention were included and further divided into the ENP group (N = 46) and the EPL group (N = 13) (Figure 1).

### Patient profiles

The patient characteristics of the two groups are listed in Table 1. Age, BMI, AMH levels, basal sex hormone levels, thyroid-stimulating hormone (TSH) levels, fT3 levels, fT4 levels, tT3 levels, tT4 levels, pbNK count before and after pregnancy, sex hormone levels, endometrial thickness, and follicle size during the period of ovulation monitoring were within the normal range. Comparisons between the two groups revealed no significant differences.

### Expression patterns of cytotoxic NK cell subsets

The gating method for NK cells is shown in Figure 2. First, leukocyte singlets were identified based on the scatterplot for FSC-H and FSC-A. Lymphocytes were gated by their FSC-A and SSC-A features, and then NK cells were gated as CD3^-^CD56^+^ cells. The gated NK cells were further classified into different subpopulations according to the CD16 expression profile. Gated pbNK cells are shown in Figure 2A and Figure 2B, and gated uNK cells are shown in Figure 2C and Figure 2D.

Each subset was compared between the two groups, and no significant differences were found Supplementary table 3.

### Expression patterns of immunoregulatory NK cell subsets

**Figure 3:**
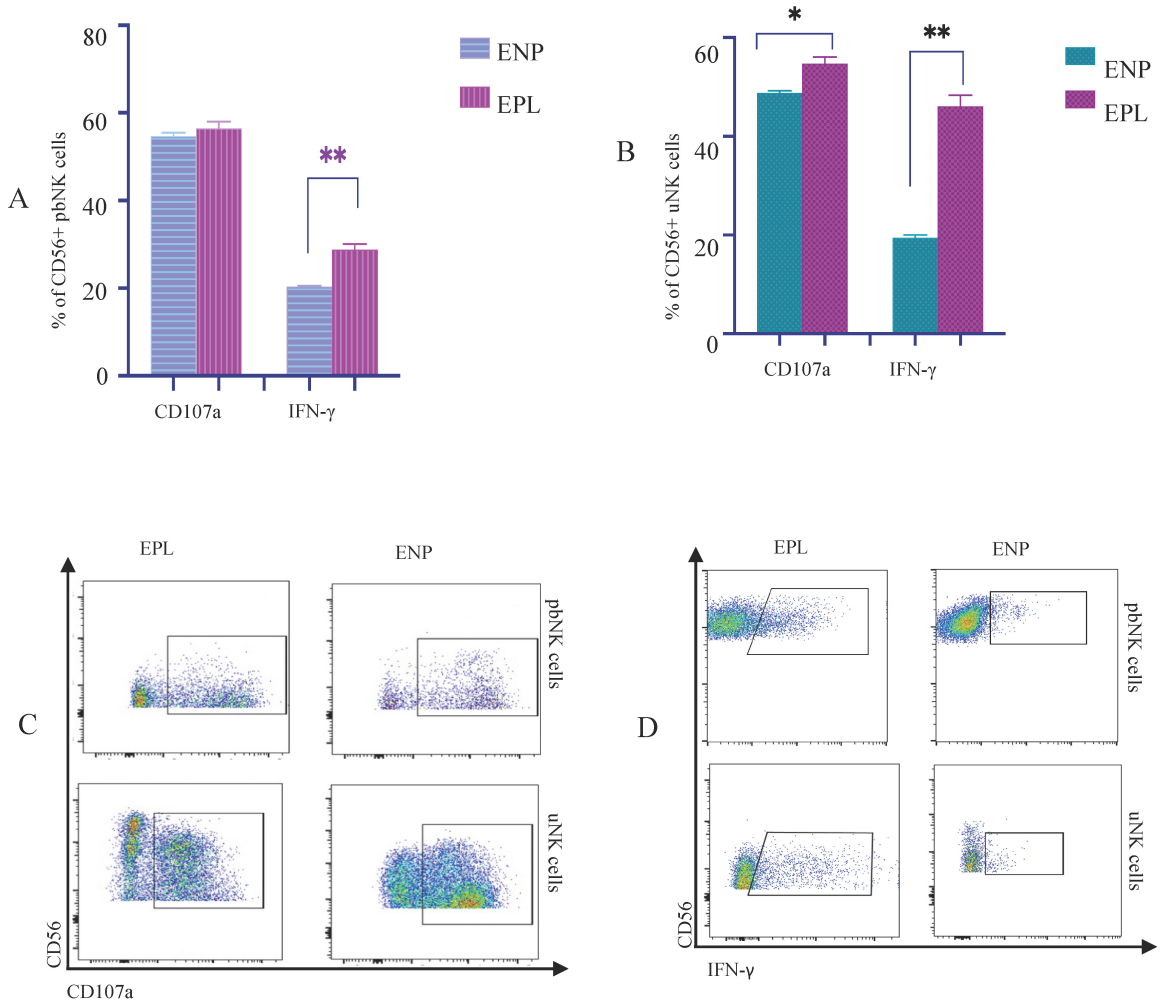
** The correlations between CD107a and cytokines on NK cells and pregnancy outcomes IFN-γ**. (A)The proportions of pbNK cells expressing CD107a and cytokines on the cell surface. (B)The proportions of uNK cells expressing the CD107a and cytokines on the cell surface. (C)Comparisons of the proportions of CD107a^+^ NK cells in pbNK cells and uNK cells between the EPL and ENP groups; (D)Comparisons of the proportions of IFN-γ NK cells in pbNK cells and uNK cells between the EPL and ENP groups. The data are expressed as the mean ± SD, and the data of the two groups were analyzed by the nonparametric Mann-Whitney U test. P<0.05 was considered to indicate a significant difference; ^*^P<0.05,^ **^P<0.01,^ ***^P<0.001,^ ****^P<0.0001.EPL: early pregnancy loss group; ENP: early normal pregnancy group. The expression level of the receptor CD107a on uNK cells in the EPL group was higher than that in the ENP group(P<0.05); The expression levels of IFN-γ in both pbNK cells and uNK cells were higher in the EPL group than in the ENP group(P<0.01).

The expression levels of degranulation markers and cytokines on pbNK cells and uNK cells in patients with different early pregnancy outcomes were compared (Figure 3A-D, Figure S4A-C). The expression levels of the CD107a receptor on uNK cells in the EPL group were higher than that in the ENP group (p = 0.02). The expression levels of the IFN-γreceptor on both pbNK cells and uNK cells in the EPL group were higher than that in the ENP group (p = 0.00 & p = 0.00).

An overview of the other immunoregulatory CD56^+^ pbNK and CD56^+^ uNK cell subsets between the ENP group and EPL group is shown in Figure S3.

### Coexpression analysis of cytotoxic and immunoregulatory NK cell subsets

**Figure 4:**
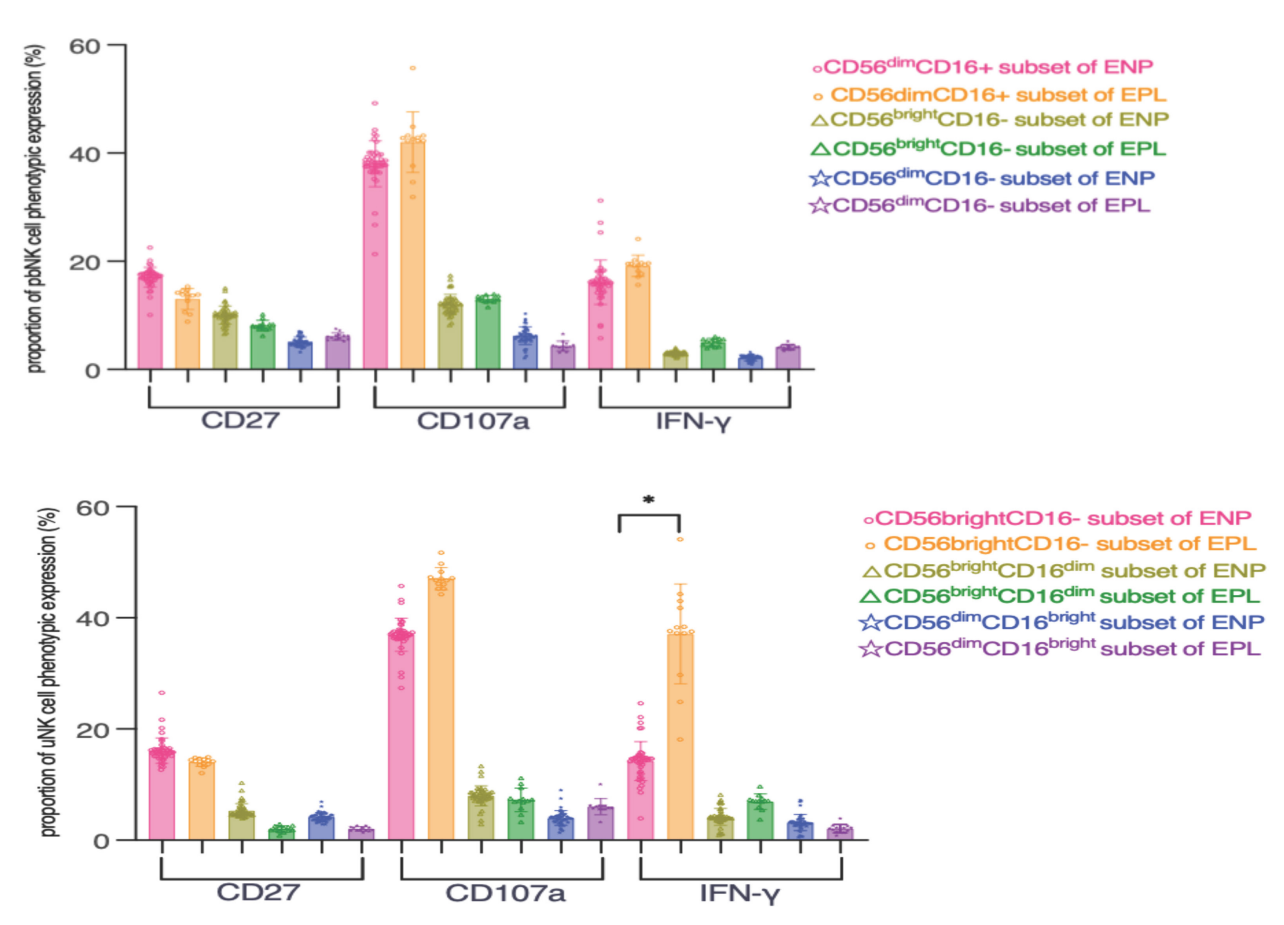
** Coexpression analysis of cytotoxic and immunoregulatory NK cell subsets during the embryo implantation window for different pregnancy outcomes.** (A) Proportion of pbNK cells expressing the indicated marker. (B) Proportion of uNK cells expressing the indicated marker. The data are expressed as the mean ± SD, and the data of the two groups were analyzed by the nonparametric Mann‒Whitney U test. P<0.05 was considered to indicate a significant difference; *P<0.05, **P<0.01, ***P<0.001, ****P<0.0001. EPL: early pregnancy loss group; ENP: early normal pregnancy group. The frequency of CD3-CD56brightIFN-γ+ uNK cells was significantly increased in the EPL group compared with the ENP group (P<0.05).

Coexpression analysis of the CD16-defined NK cell subsets with the identified significant immunoregulatory subsets was further performed, and the frequency of CD3^-^CD56^bright^IFN-γ^+^ uNK cells was significantly increased in the EPL group compared with the ENP group (p = 0.03) (Figure 4A-B).

### Multivariable regression analysis for pregnancy loss

We used multivariable regression analysis to analyze the associations between the potential predictors and pregnancy outcomes. Among all the factors, the frequency of CD3^-^CD56^+^ IFN-γ^+^ uNK cells individually was positively related to early pregnancy loss. The details are shown in Table 2.

## Discussion

To the best of our knowledge, this is the first study on the correlations between the functional features of NK cells during the embryo implantation window and pregnancy outcomes in uRPL. We found that an increase in the frequency of CD3^-^CD56^+^IFN-γ^+^ uNK cells was associated with the recurrence of pregnancy loss in uRPL.

The inclusion and exclusion criteria for patients with uRPL in this study were more stringent than those in previous studies. This was achieved by using currently available investigations to exclude maternal and paternal factors known to be associated with RPL. Currently pregnant patients who received therapeutic interventions in early pregnancy supervision due to the consequent medical burden were further excluded. The nuchal translucency ultrasound scan and Down's syndrome screening results for patients with successful pregnancy outcomes were normal, indicating normal fetal development during the first trimester, which makes the study rigorous and convincing.

Peripheral blood and endometrial samples collected during the implantation window (i.e., mid-luteal phase) were obtained, and lymphocytes were isolated to construct an *in vitro* trophoblast-lymphocyte coculture system mimicking the environment necessary for embryo implantation. The HTR-8/SVneo trophoblast cell line has been widely used in research for a long time, and previous studies have confirmed that many HLA-I and especially HLA-C molecules are abundantly expressed on the HTR-8/SVneo trophoblast cell line 24, 25. The HTR-8/SVneo cell line contains a heterogeneous population of trophoblast and mesenchymal cells that exhibit features relatively similar to the typical characteristics of normal early gestational trophoblast cells 26. This study simulated the pregnancy environment at the maternal-fetal interface, which is innovative and makes this approach a more convincing way to explore the role of NK cells in uRPL.

The proportions of the dominant subsets in the peripheral blood and endometrium were consistent with those reported in earlier researches 17, 18, indicating the feasibility of the approach in this study for NK cell isolation and that pbNK cells played a predominantly cytotoxic role. However, uNK cells played a predominantly immunomodulatory role during embryo implantation in uRPL. Based on the finding that the proportions of pbNK and uNK cell subsets defined by the same phenotype were not significantly different between different pregnancy outcomes, it was speculated that the CD16-defined cytotoxic subsets do not play a significant role during embryo implantation in uRPL. Therefore, we investigated the role of immunoregulatory NK cell subsets in uRPL.

NK cells are a major source of interferon (IFN)-γ during embryo implantation, IFN-γ contributes to initiation of uterine vascular modification, decidual integrity, and uNK cell maturation during normal murine pregnancy^27^. NK cells can be stimulated to secrete IFN-γ in both receptor-mediated and cytokine-mediated pathways, and uNK cells primarily produce cytokines including IFN-γ rather than performing cytotoxicity. As previously described, IFN-γ was produced in response to the cooperative stimulation by NK cell surface receptors as well as cytokines^28^. IFN-γ promoted the uNK cell maturation, IFN-γ promoted the uNK cell maturation, and IFN-γ produced by NK cells was confirmed to be promote the remodeling of the uterine spiral arteries during the decidualization of pregnancy and to a key factor in maintaining a successful pregnancy^27^, ^29^.

CD107a staining is a proxy for degranulation, and it acts as a reliable measure of overall uNK activation 30. While CD107a degranulation in NK cells is a key indicator of their cytotoxic activity, uNK cells primarily exhibit immunomodulatory functions (e.g., IFN-γ secretion) rather than cytotoxicity during implantation 31, 32. The role of IFN-γ secreted by NK cells in pregnancy is well studied, and it is well known that IFN-γ promotes embryo implantation. However, elevated production of this cytokine is detrimental to embryo survival 33, and IFN-γ can prevent implantation and inhibit pregnancy maintenance at the preimplantation stage 34, 35. The mechanisms are as follows: i) the expression of proteins required for embryo adhesion was confirmed to be altered in response to IFN-γ 36; and ii) IFN-γ inhibited the secretion of the cytokine GM-CSF, which plays a key role in promoting blastocyst development and differentiation, in the reproductive tract epithelial cells of mice and humans 37. This observation is consistent with the established functional heterogeneity of decidual NK cells, which are predominantly CD56bright, possess low cytotoxic potential, and primarily function through cytokine production rather than direct killing 38. Importantly, different dNK subsets exhibit distinct functional capacities shaped by the local decidual microenvironment and can secrete cytokines such as GM-CSF that influence trophoblast migration and placental development 38. Our finding that the increase in IFN-γ production is specifically localized to the CD56^bright^CD16⁻ subset underscores the importance of dissecting these functional subpopulations, as a shift in the balance between them, rather than a global change in all uNK cells, may underlie the pathological effects observed in uRPL.

In the present study, we found that increased expression of IFN-γ in both pbNK cells and uNK cells was related to the occurrence of pregnancy loss in uRPL. In addition, the frequency of CD56brightCD16-IFN-γ+ uNK cells was significantly increased in patients with pregnancy loss, suggesting that an increased population of IFN-γ+ NK cells may play a role in uRPL. These findings suggest that elevated IFN-γ production by NK cells, particularly within the CD56brightCD16⁻ uNK subset, is associated with uRPL. The specific enrichment of this phenotype during the implantation window points to a potential role for IFN-γ+ uNK cells in the pathogenesis of uRPL, though whether this represents a primary dysfunction or a secondary response to other implantation defects remains to be determined.

Cytotoxic responses may be secondary to trophoblast stress signals 39, whereas chronic IFN-γ production drives sustained inflammation linked to implantation failure 40. While CD107a reliably marks uNK activation, its elevation in pregnancy loss may reflect that cytotoxic degranulation occur after initial implantation failure, making it a consequence rather than a cause. This may explain why CD107a+ uNK frequency, though higher in pregnancy loss, did not independently predict outcomes, suggesting CD107a+ uNKs are a secondary marker rather than a primary driver of pregnancy loss. pbNK-derived IFN-γ may be buffered by systemic regulatory mechanisms, whereas uterine IFN-γ acts locally on trophoblasts and spiral arteries. Peripheral IFN-γ increases may be insufficient to disrupt pregnancy without concurrent uterine inflammation during embryo implantation. The multivariate analysis likely excluded CD107a+ uNK and peripheral IFN-γ+ NK cells due to: a) IFN-γ+ uNK cells may overshadow other variables by directly impair trophoblast invasion 27, 41, 42; b) IFN-γ+ uNK cells may contribute to pregnancy failure by disrupting the angiogenic balance at the maternal-fetal interface 43.

Our data suggest that elevated frequencies of CD3-CD56+IFN-γ+ uNK cells at the maternal-fetal interface during the implantation window may serve as a potential predictor for subsequent pregnancy outcomes (≤ 12 weeks) in women with unexplained recurrent pregnancy loss (uRPL).

This study has several limitations that should be acknowledged. First, the lack of a healthy control group using endometrial tissue from normal pregnancies during the implantation window represents an inherent challenge in human reproductive research. Due to the invasive nature of the procedure and substantial ethical barriers in obtaining mid-luteal phase endometrial samples from healthy volunteers, most previous studies investigating endometrial NK cells in RPL have relied on decidual tissues from pregnancy loss cases or animal models, which is difficult to determine whether changes in dNK cells are causes or consequence of pregnancy loss. For controls, they utilize tissue from women with prior live births undergoing elective termination of unplanned pregnancies (often presumed "healthy") as controls, which introduces potential selection bias since these women typically don't undergo comprehensive RPL screening and the embryonic viability remains unknown (i.e., some conceptuses might have been destined for pregnancy loss), thus this may systematically include cases with subclinical reproductive pathologies. While we addressed this by using pregnancy outcomes as endpoints (with RPL patients achieving first-trimester viability serving as controls), future studies should explore ethical approaches to incorporate true healthy controls when possible. Additionally, despite our prospective collection of samples prior to pregnancy outcome, the fundamental question of causality remains. Our study design identifies an association between elevated IFN-γ+ uNK cells and subsequent pregnancy loss, but it cannot definitively prove whether this immune phenotype is a primary cause of the loss or a consequence of a previously compromised implantation process. Future studies using animal models or longitudinal sampling throughout early gestation may help elucidate the temporal sequence of these events.

Second, limitations related to follow-up and sample size. Our follow-up strategy introduced unavoidable complexity. While uRPL is uncommon and recruiting patients for comprehensive diagnostic testing is challenging, a feasible approach to expand our cohort is to closely follow all consented patients to increase both the normal pregnancy and pregnancy loss subgroups. However, our clinical experience confirmed that pregnancy follow-up beyond 12 weeks substantially increased the probability of patients initiating various treatments. Driven by anxiety about recurrent pregnancy loss, most enrolled patients received heterogeneous interventions across different institutions during follow-up. For patients who remained untreated, dropout rates increased significantly beyond 12 weeks of gestation, likely because those who passed this critical risk period were less motivated to continue follow-up (patient-initiated attrition). This natural loss to follow-up reflects the real-world clinical challenge of maintaining longitudinal engagement in RPL populations after perceived 'safety milestones'. These factors introduce significant heterogeneity and increase potential bias that that may confound results and complicates mechanistic interpretations. Although we mitigated this by using early pregnancy outcomes (≤ 12 weeks) as endpoints to reduce dropout rates and intervention variability, future work should seek multicenter collaborations to maintain the 12-week endpoint to minimize confounders as well as work on larger population or ideally track patients through live birth while rigorously documenting all interventions. Furthermore, although our sample size calculation was met, we fully acknowledge that the modest number of patients in the EPL group (n=13) represents a major and important limitation. This small sample size is a primary concern, as it inevitably limited the statistical power of our multivariable analysis and precluded more detailed subgroup analyses. Therefore, the results from the regression model, particularly the identification of IFN-γ⁺ uNK cells as an independent predictor, should be interpreted as no more than exploratory and require urgent validation in larger, independent cohorts.

Third, limitations related to the *in vitro* model. The maternal-fetal interface involves prolonged crosstalk *in vivo*, while our 6-hour co-culture system captured acute NK-trophoblast interactions relevant to the implantation window, we acknowledge that it does not fully recapitulate the complexity of the *in vivo* maternal-fetal interface, and that longer durations might reveal additional signaling dynamics. Future studies could employ time-course experiments to dissect temporal changes in NK cell function. Although our 6-hour assay aligns with the acute diagnostic need (predicting early pregnancy outcomes), extended cultures might better model later gestational events (e.g., spiral artery remodeling). However, such designs must balance physiological relevance against technical artifacts (e.g., cell stress in prolonged *in vitro* cultures). While we prioritized detecting initial NK activation (CD107a/IFN-γ) with minimal artifacts, alternative methods (e.g., multiplex marker profiling over 24h) could complement our findings.

Fourth, limitations related to etiologic exclusion and statistical confounding. While comprehensive etiologic examinations were performed to exclude common causes of RPL, we acknowledge that gestational trophoblastic diseases (GTD) such as hydatidiform mole were not ruled out by the gold standard method of histologic examination of pregnancy loss tissue 44. However, it is important to note that all patients in our cohort underwent serial ultrasound evaluations during the implantation window and early pregnancy, which showed no evidence of the classic ultrasonographic features of hydatidiform mole, such as diffuse cystic spaces within the placenta, irregularity of the decidua, or absence of an embryo/fetus 44. While ultrasound is less definitive than histology for excluding GTD, particularly in early pregnancy, the absence of suspicious findings in our cohort reduces the likelihood that undiagnosed molar pregnancies confound our results. Nevertheless, future studies incorporating post-loss tissue analysis would provide even more definitive exclusion of such pathologies. Additionally, although our multivariable model adjusted for key clinical and immunological factors (maternal age, BMI, frequencies of IFN-γ⁺ and CD107a⁺ uNK cells, and history of previous pregnancy loss), we cannot exclude the possibility of residual confounding from unmeasured variables. These may include detailed prior pregnancy history (e.g., number and gestational age of previous losses), subtle hormonal fluctuations, or undetected endometrial characteristics, all of which could potentially influence both NK cell function and pregnancy outcome. Future studies with more comprehensive data collection should consider these factors in their models.

Despite these constraints, our study provides novel insights by establishing a trophoblast-lymphocyte co-culture system using implantation-phase samples from uRPL patients—a methodological advance over prior decidua-based or animal models. Further research should: 1 validate findings in larger cohorts with standardized follow-up protocols, 2 incorporate live birth endpoints where feasible, 3 explore non-invasive biomarkers to overcome tissue acquisition challenges, and^4^ following its identification as a significant risk factor in this study, determine the optimal prognostic cut-off for IFN-γ+ uNK cells in future large-scale validation studies (e.g., using ROC analysis in adequately powered cohorts), as the current exploratory sample size does not support stable ROC estimation, including its role in multi-factor predictive models. In this study, the early pregnancy outcome (≤12 weeks) was used as an endpoint, and patients without identifiable causes of RPL and with no additional pregnancy loss at the end of the first trimester were included in the normal control group. This paper provides unprecedented knowledge on the functional features of NK cells during the embryo implantation window associated with pregnancy outcomes in uRPL.

We hope that future research will help elucidate the exact underlying process involving IFN-γ+ NK cells in the pathogenesis of uRPL and that exploring IFN-γ-targeted therapy may help revolutionize the way uRPL is treated in the future, resulting in rectifying the gray market with immunotherapies and reducing overtreatment in primary level hospitals. The identified association between elevated IFN-γ+ uNK cells and recurrent pregnancy loss suggests the potential therapeutic value of modulating this pathway. By targeting the JAK-STAT pathway, JAK inhibitors achieve broad suppression of key pro-inflammatory cytokines, including IFN-γ 45, 46. This mechanism provides a strong theoretical basis for using them to modulate the detrimental effects of elevated IFN-γ at the maternal-fetal interface 45, 47. Another promising approach involves targeting the upstream activator NKG2D on uNK cells; studies indicate that blockading the NKG2D receptor with antibodies or soluble ligands can prevent the uNK cells from becoming activated and reduces or eliminates the production of IFN-γ 48, 49, thereby mitigating its inhibitory effects on invasion. Future research should prioritize these more targeted strategies to determine optimal timing and dosage in preclinical models, aiming to correct the pathological imbalance of IFN-γ while preserving its essential physiological functions in early gestation.

## Conclusions

Higher frequencies of CD3-CD56+IFN-γ+ NK cells in the implantation window are associated with increased risk of pregnancy loss (≤ 12 weeks) in uRPL patients, suggesting a potential biomarker that requires validation in larger studies.

## Supplementary Material

Supplementary figures and tables.

## Figures and Tables

**Table 1:** Baseline characteristics of patients with different pregnancy outcomes.

	ENP (N = 46)		EPL (N = 13)	P value
**Age**	31.9.4±4.3		33.1±2.8	0.348
**BMI (kg/m^2^) before pregnancy**	21.2±2.3		21.5±2.7	0.854
**AMH (ng/mL)**	3.4±1.1		3.3±1.7	0.140
**Ovarian function**				
FSH (IU/L)	5.5±1.7		5.7±3.3	0.092
LH (IU/L)	3.6±1.6		3.5±0.9	0.145
Estradiol (pg/mL)	36.3±8.7		37.6±11.9	0.432
PRL (ng/mL)	30.6±9.1		29.7±12.3	0.619
T (ng/mL)	0.4±0.2		0.3±0.2	0.517
P (ng/mL)	0.3±0.0		0.2±0.1	0.781
**Thyroid function (first trimester)**				
TSH (μIU/mL)	1.9±0.7		2.0±0.9	0.281
fT_3_ (pmol/L)	4.3±1.1		4.5±0.9	0.873
fT_4_ (pmol/L)	16.9±6.2		16.2±5.7	0.451
**pbNK count in lymphocytes (nonpregnant)**				
CD3^-^CD56^+^ NK cells	16.8±2.1		17.0±4.3	0.116
CD3^-^CD56^+^CD16^+^ NK cells	15.1±4.7		14.9±5.5	0.778
CD3^-^CD56^+^CD16^-^ NK cells	6.5±2.6		6.1±2.3	0.472
**pbNK count in lymphocytes (pregnant)**				
CD3^-^CD56^+^ NK cells	17.5±4.4		18.1±6.7	0.378
CD3^-^CD56^+^CD16^+^ NK cells	15.9±3.2		16.1±4.2	0.923
CD3^-^CD56^+^CD16^-^ NK cells	6.6±0.2		6.5±1.6	0.612
**FSH level on LH surge day (IU/L)**	10.4±3.1		11.5±2.4	0.166
**LH surge level (IU/L)**	52.6±7.7		49.8±9.5	0.738
**Estradiol level on LH surge day (pg/mL)**	256.5±16.4		249.3±22.8	0.555
**Progesterone level on LH surge day (ng/mL)**	0.8±0.1		0.7±0.1	0.471
**Endometrial thickness on LH surge day (pg/mL)**	8.7±2.2		8.8±1.9	0.972
**Follicle diameter on LH surge day (mm)**	19.1±0.5		19.0±0.0	0.643
**Estradiol level on LH surge +7 day (pg/mL)**	117.8±36.7		111.2±25.9	0.882
**Progesterone level on LH surge +7 day (ng/mL)**	21.7±4.5		20.1±1.1	0.325
**Endometrial thickness on LH surge +7 day (mm)**	9.0±1.1		8.9±0.6	0.112

EPL: early pregnancy loss group; ENP: early normal pregnancy group; BMI: body mass index; FSH: follicle-stimulating hormone; fT3: free thyroid thyronine; fT4: free thyroxine; LH: luteinizing hormone; TSH: thyroid-stimulating hormone. The data are expressed as the mean ± SD, and the data of the two groups were analyzed by the nonparametric Mann-Whitney U test.

**Table 2:** Association between the frequency of NK cells and pregnancy outcomes.

Characteristic	Early pregnancy loss		
	OR^*^	95% CI	p value
CD27^+^ uNk	0.891	0.782-1.070	0.850
CD107a^+^ uNk	1.483	0.981-1.647	0.456
IFN-γ^+^ pbNK	2.140	0.885-4.400	0.088
IFN-γ^ +^ uNK	1.198	1.038-1.583	0.008

*Adjusted for maternal age, BMI, and history of previous pregnancy loss. uNk: uterine Natural Killer cells; pbNk: peripheral blood Natural Killer cells; CI: confidence interval. P<0.05 was set as the threshold to indicate a statistically significant difference. Multivariable linear regression was used to analyze the association between the frequency of functional NK cells and early pregnancy loss.

## Data Availability

Data will be made available on request.

## References

[1] Youssef A, Lashley L, Dieben S, Verburg H, van der Hoorn ML (2020). Defining recurrent pregnancy loss: associated factors and prognosis in couples with two versus three or more pregnancy losses. Reprod Biomed Online.

[2] ESHRE (2018). ESHRE guideline: recurrent pregnancy loss. Human Reproduction Open.

[3] Practice Committee of the American Society for Reproductive Medicine (2013). Definitions of infertility and recurrent pregnancy loss: a committee opinion. Fertility and Sterility.

[4] Turesheva A, Aimagambetova G, Ukybassova T, Marat A, Kanabekova P, Kaldygulova L (2023). Recurrent Pregnancy Loss Etiology, Risk Factors, Diagnosis, and Management. Fresh Look into a Full Box. J Clin Med.

[5] Sojka DK, Yang L, Plougastel-Douglas B, Higuchi DA, Croy BA, Yokoyama WM (2018). Cutting Edge: Local Proliferation of Uterine Tissue-Resident NK Cells during Decidualization in Mice. J Immunol.

[6] Sojka DK, Yang L, Yokoyama WM (2019). Uterine Natural Killer Cells. Front Immunol.

[7] Wira CR, Rodriguez-Garcia M, Patel MV (2015). The role of sex hormones in immune protection of the female reproductive tract. Nat Rev Immunol.

[8] Feyaerts D, Kuret T, van Cranenbroek B, van der Zeeuw-Hingrez S, van der Heijden OWH, van der Meer A (2018). Endometrial natural killer (NK) cells reveal a tissue-specific receptor repertoire. Hum Reprod.

[9] Andreotti JP, Paiva AE, Prazeres P, Guerra DAP, Silva WN, Vaz RS (2018). The role of natural killer cells in the uterine microenvironment during pregnancy. Cell Mol Immunol.

[10] Vento-Tormo R, Efremova M, Botting RA, Turco MY, Vento-Tormo M, Meyer KB (2018). Single-cell reconstruction of the early maternal-fetal interface in humans. Nature.

[11] Kuon RJ, Weber M, Heger J, Santillan I, Vomstein K, Bar C (2017). Uterine natural killer cells in patients with idiopathic recurrent miscarriage. Am J Reprod Immunol.

[12] El-Azzamy H, Dambaeva SV, Katukurundage D, Salazar Garcia MD, Skariah A, Hussein Y (2018). Dysregulated uterine natural killer cells and vascular remodeling in women with recurrent pregnancy losses. Am J Reprod Immunol.

[13] Michimata T, Ogasawara MS, Tsuda H, Suzumori K, Aoki K, Sakai M (2002). Distributions of endometrial NK cells, B cells, T cells, and Th2/Tc2 cells fail to predict pregnancy outcome following recurrent abortion. Am J Reprod Immunol.

[14] Shimada S, Kato EH, Morikawa M, Iwabuchi K, Nishida R, Kishi R (2004). No difference in natural killer or natural killer T-cell population, but aberrant T-helper cell population in the endometrium of women with repeated miscarriage. Hum Reprod.

[15] Zhao Y, Chen X, Zhang T, Chan LKY, Liu Y, Chung JP (2020). The use of multiplex staining to measure the density and clustering of four endometrial immune cells around the implantation period in women with recurrent miscarriage: comparison with fertile controls. J Mol Histol.

[16] Lapides L, Klein M, Belusakova V, Csobonyeiova M, Varga I, Babal P (2022). Uterine Natural Killer Cells in the Context of Implantation: Immunohistochemical Analysis of Endometrial Samples from Women with Habitual Abortion and Recurrent Implantation Failure. Physiol Res.

[17] Von Woon E, Greer O, Shah N, Nikolaou D, Johnson M, Male V (2022). Number and function of uterine natural killer cells in recurrent miscarriage and implantation failure: a systematic review and meta-analysis. Hum Reprod Update.

[18] Yuzen D, Arck PC, Thiele K (2022). Tissue-resident immunity in the female and male reproductive tract. Semin Immunopathol.

[19] Diaz-Hernandez I, Alecsandru D, Garcia-Velasco JA, Dominguez F (2021). Uterine natural killer cells: from foe to friend in reproduction. Hum Reprod Update.

[20] Di Simone N, Caliandro D, Castellani R, Ferrazzani S, Caruso A (2000). Interleukin-3 and human trophoblast: *in vitro* explanations for the effect of interleukin in patients with antiphospholipid antibody syndrome. Fertil Steril.

[21] Fu B, Zhou Y, Ni X, Tong X, Xu X, Dong Z (2017). Natural Killer Cells Promote Fetal Development through the Secretion of Growth-Promoting Factors. Immunity.

[22] Huhn O, Chazara O, Ivarsson MA, Retiere C, Venkatesan TC, Norman PJ (2018). High-Resolution Genetic and Phenotypic Analysis of KIR2DL1 Alleles and Their Association with Pre-Eclampsia. J Immunol.

[23] Johnsen GM, Storvold GL, Drabbels JJM, Haasnoot GW, Eikmans M, Spruyt-Gerritse MJ (2018). The combination of maternal KIR-B and fetal HLA-C2 is associated with decidua basalis acute atherosis in pregnancies with preeclampsia. J Reprod Immunol.

[24] Abbas Y, Turco MY, Burton GJ, Moffett A (2020). Investigation of human trophoblast invasion *in vitro*. Hum Reprod Update.

[25] Lv H, Zhou Q, Li L, Wang S (2021). HLA-C promotes proliferation and cell cycle progression in trophoblast cells. J Matern Fetal Neonatal Med.

[26] Msheik H, Azar J, El Sabeh M, Abou-Kheir W, Daoud G (2020). HTR-8/SVneo: A model for epithelial to mesenchymal transition in the human placenta. Placenta.

[27] Nurzadeh M, Ghalandarpoor-Attar SM, Ghalandarpoor-Attar SN, Rabiei M (2023). The Role of Interferon (IFN)-gamma in Extravillous Trophoblast Cell (EVT) Invasion and Preeclampsia Progression. Reprod Sci.

[28] Yang X, Tian Y, Zheng L, Luu T, Kwak-Kim J (2022). The Update Immune-Regulatory Role of Pro- and Anti-Inflammatory Cytokines in Recurrent Pregnancy Losses. Int J Mol Sci.

[29] Yockey LJ, Iwasaki A (2018). Interferons and Proinflammatory Cytokines in Pregnancy and Fetal Development. Immunity.

[30] Alter G, Malenfant JM, Altfeld M (2004). CD107a as a functional marker for the identification of natural killer cell activity. J Immunol Methods.

[31] Lash GE, Robson SC, Bulmer JN (2010). Review: Functional role of uterine natural killer (uNK) cells in human early pregnancy decidua. Placenta.

[32] Zhou J, Yan P, Ma W, Li J (2025). Cytokine modulation and immunoregulation of uterine NK cells in pregnancy disorders. Cytokine Growth Factor Rev.

[33] Robertson SA, Chin PY, Femia JG, Brown HM (2018). Embryotoxic cytokines-Potential roles in embryo loss and fetal programming. J Reprod Immunol.

[34] Pinheiro MB, Martins-Filho OA, Mota AP, Alpoim PN, Godoi LC, Silveira AC (2013). Severe preeclampsia goes along with a cytokine network disturbance towards a systemic inflammatory state. Cytokine.

[35] Bacenkova D, Trebunova M, Cizkova D, Hudak R, Dosedla E, Findrik-Balogova A (2022). *In vitro* Model of Human Trophoblast in Early Placentation. Biomedicines.

[36] Fontana V, Choren V, Vauthay L, Calvo JC, Calvo L, Cameo M (2004). Exogenous interferon-gamma alters murine inner cell mass and trophoblast development. Effect on the expression of ErbB1, ErbB4 and heparan sulfate proteoglycan (perlecan). Reproduction.

[37] Sharkey DJ, Glynn DJ, Schjenken JE, Tremellen KP, Robertson SA (2018). Interferon-gamma inhibits seminal plasma induction of colony-stimulating factor 2 in mouse and human reproductive tract epithelial cells. Biol Reprod.

[38] Sharkey AM, Xiong S, Kennedy PR, Gardner L, Farrell LE, Chazara O (2015). Tissue-Specific Education of Decidual NK Cells. J Immunol.

[39] Tan HX, Yang SL, Li MQ, Wang HY (2020). Autophagy suppression of trophoblast cells induces pregnancy loss by activating decidual NK cytotoxicity and inhibiting trophoblast invasion. Cell Commun Signal.

[40] Rezaei M, Moghoofei M (2024). The role of viral infection in implantation failure: direct and indirect effects. Reprod Biol Endocrinol.

[41] Murphy SP, Tayade C, Ashkar AA, Hatta K, Zhang J, Croy BA (2009). Interferon gamma in successful pregnancies. Biol Reprod.

[42] Hu Y, Dutz JP, MacCalman CD, Yong P, Tan R, von Dadelszen P (2006). Decidual NK cells alter *in vitro* first trimester extravillous cytotrophoblast migration: a role for IFN-gamma. J Immunol.

[43] Li ZY, Chao HH, Liu HY, Song ZH, Li LL, Zhang YJ (2014). IFN-gamma induces aberrant CD49b(+) NK cell recruitment through regulating CX3CL1: a novel mechanism by which IFN-gamma provokes pregnancy failure. Cell Death Dis.

[44] Libretti A, Longo D, Faiola S, De Pedrini A, Troia L, Remorgida V (2023). A twin pregnancy with partial hydatidiform mole and a coexisting normal fetus delivered at term: A case report and literature review. Case Rep Womens Health.

[45] Shao T, Leung PSC, Zhang W, Tsuneyama K, Ridgway WM, Young HA (2022). Treatment with a JAK1/2 inhibitor ameliorates murine autoimmune cholangitis induced by IFN overexpression. Cell Mol Immunol.

[46] Zavoriti A, Miossec P (2025). Understanding the effects of Janus kinase inhibitors on the cardiovascular system in comparison to main biological DMARDs in rheumatoid arthritis. Autoimmun Rev.

[47] Liu H, Wang W, Liu C (2021). Increased expression of IFN-gamma in preeclampsia impairs human trophoblast invasion via a SOCS1/JAK/STAT1 feedback loop. Exp Ther Med.

[48] Steigerwald J, Raum T, Pflanz S, Cierpka R, Mangold S, Rau D (2009). Human IgG1 antibodies antagonizing activating receptor NKG2D on natural killer cells. MAbs.

[49] Carayannopoulos LN, Barks JL, Yokoyama WM, Riley JK (2010). Murine trophoblast cells induce NK cell interferon-gamma production through KLRK1. Biol Reprod.

[50] Niwei Yan, Pingyin Lee, Huiying Jie, Canquan Zhou, Yuan Yuan The associations of natural killer cell functions during the embryo implantation window with pregnancy outcomes in women for whom the number of peripheral blood natural killer cells cannot be applied as a therapeutic index for immunological abnormalities in unexplained recurrent pregnancy loss. https://www.researchsquare.com/article/rs-3000344/v1.

