# Supplementary material for: IFN-γ+ NK cells as a potential predictor of pregnancy loss in unexplained recurrent pregnancy loss
Source: Int J Med Sci. 2026 May 29;23(7):2302–14. doi: 10.7150/ijms.121925 (PMC13280750; doi:10.7150/ijms.121925)
Supplement: Supplementary file 1 — Supplementary figures and tables. [file ijmsv23p2302s1.pdf]

Supplementary Figures

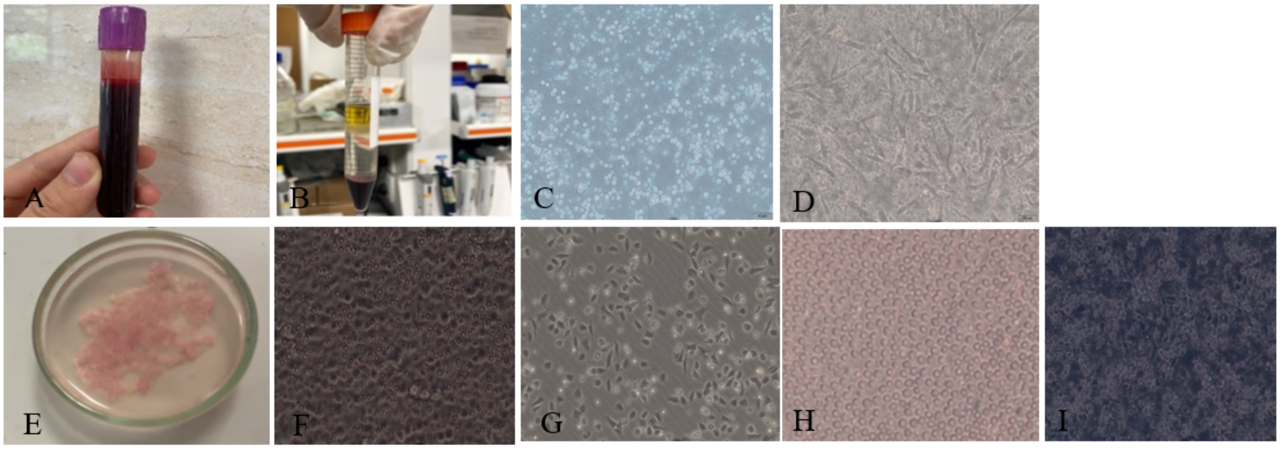

Supplementary Figure 1. Processing of peripheral blood and endometrial samples. (A) Fresh blood sample obtained before endometrial biopsy. (B) Lymphocytes (cloudy layer) isolated by density gradient centrifugation after Ficoll separation. (C) Micrograph of peripheral blood lymphocytes. (D) Peripheral blood lymphocyte-HTR-8/SVneo trophoblast cell coculture. (E) Digestion of endometrial tissue pieces. (F) Microscopic view of an endometrial suspension including endometrial stromal cells and lymphocytes obtained by isolating glandular epithelial cells with a 40-µm sterile filter after digestion. (H) Endometrial stromal cells isolated by adherent culture. (H) Microscopic view of endometrial lymphocytes after isolating endometrial stromal cells. (I) Endometrial lymphocyte-HTR-8/SVneo trophoblast cell coculture.

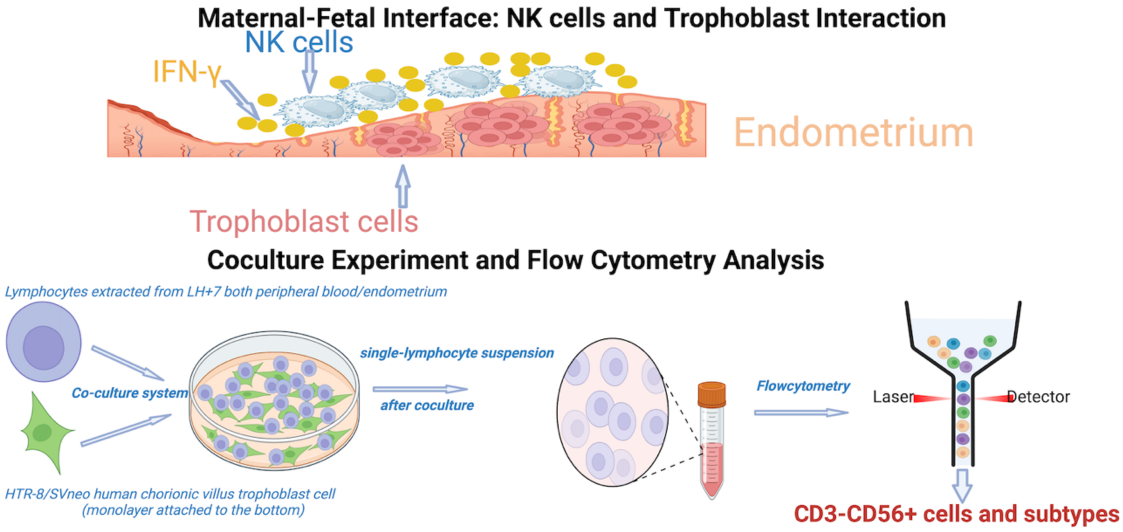

Supplementary Figure 2. Experimental setup for the HTR-8/SVneo-lymphocyte co-culture model.

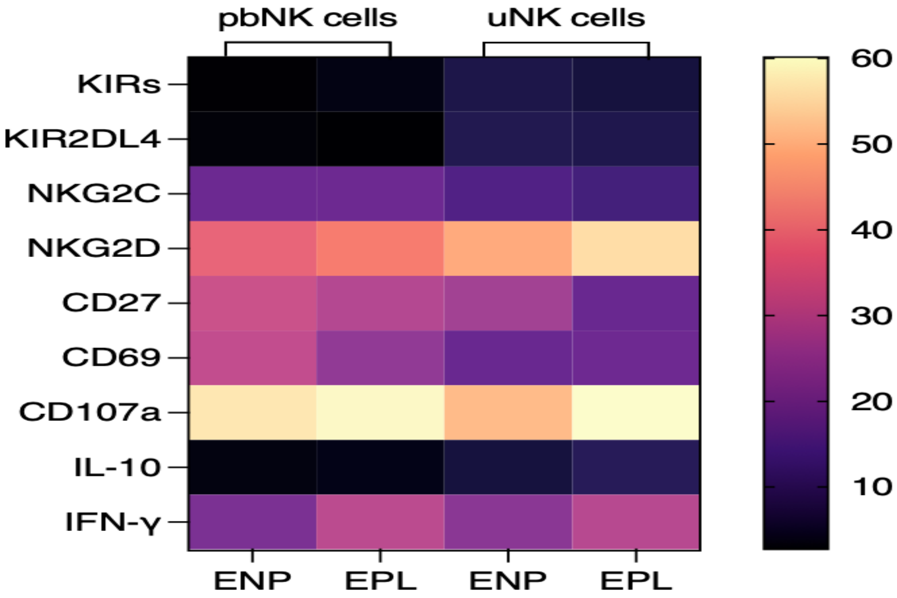

Supplementary figure 3. Expression patterns of immunoregulatory CD56^{+} pbNK and uNK cell subsets during the embryo implantation window compared among uRPL patients with different pregnancy outcomes. The expression level of the CD27 receptor on uNK cells in the EPL group was lower than that in the ENP group ($P<0.01$). The expression level of the receptor CD107a on uNK cells in the EPL group was higher than that in the ENP group ($P<0.01$). The expression levels of IFN-γ in both pbNK cells and uNK cells were higher in the EPL group than in the ENP group ($P<0.0001$). EPL: early pregnancy loss group; ENP: early normal pregnancy group.

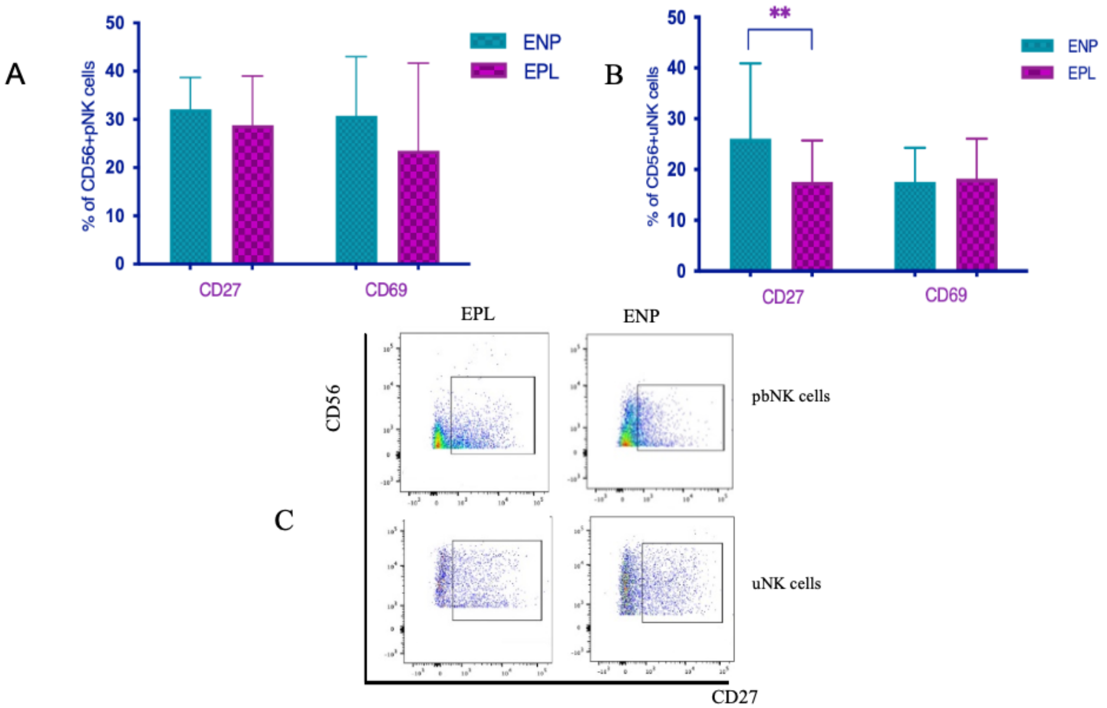

Supplementary figure 4. The correlations between signaling-regulated receptors on NK cells and pregnancy

(A) The proportions of pbNK cells expressing the indicated signaling-regulated receptors on the cell surface. (B) The proportions of uNK cells expressing the indicated signaling-regulated receptors on the cell surface. (C) Comparisons of the proportions of CD27+ NK cells in pbNK cells and uNK cells between the EPL and ENP groups. The data are expressed as the mean ± SD, and the data of the two groups were analyzed by the nonparametric Mann–Whitney U test. P<0.05 was considered to indicate a significant difference; *P<0.05, **P<0.01, ***P<0.001, ****P<0.0001. EPL: early pregnancy loss group; ENP: early normal pregnancy group. The expression level of the CD27 receptor on uNK cells in the EPL group was lower than that in the ENP group (P<0.01).

Supplementary tables

Supplementary table 1. NK cell surface receptor and secreted cytokine information

Receptor	Structure	Ligand	Uncoupling protein	Main functions and effects on reproduction
CD16	Ig superfamily	Fc	CD3ζ and FcεRIγ	Cytotoxic NK cell subsets. Performs a killing function through ADCC
KIR2DL2/L3/S2(KIRs)	KIR receptor	HLA-C	SHP-1 and SHP-2	Cytotoxicity-regulated NK cell subsets. Inhibitory receptor; Participate in pregnancy immune tolerance
KIR2DL4	KIR receptor	HLA-G	FcεRIγ	Cytotoxicity-regulated NK cell subsets. Negative regulation of NK cytotoxicity; Positive regulation of cytokine secretion produced by NK cells; Participate in pregnancy immune tolerance
NKG2C	killer cell lectin-like receptor	HLA-E	Complex of CD94 and DAP12	Cytotoxicity-regulated NK cell subsets and play a defensive role during viral infection in mice
NKG2D	natural cytotoxicity receptor	MICA; MICB; ULBP	DAP-10	Cytotoxicity-regulated NK cell subsets. Activating receptor: positive regulation of NK cell cytotoxicity, negative regulation of NK cell chemotaxis, expressed on endometrial NK cells in pregnant mice
CD27	tumor necrosis factor receptor superfamily	None	None	Signaling-regulated NK cell subsets. Negative regulation of apoptotic process; positive regulation of signaling supporting cytokine production, proliferation and functional regulation; CD27^{low} expression can regulate the production of a large number of IFN-γ-related factors and improve pregnancy loss in mice
CD69	C-type lectin superfamily	None	None	Signaling-regulated NK cell subsets. Regulates the binding of calcium ions and carbohydrates, and trans-signaling receptor activity; positive regulation of NK cell cytotoxicity, proliferation, and IFN-γ production; direct induction of NK cell cytolytic activity in the decidua vera
CD107a		None	None	NK cell degranulation marker, identifies NK cells that have been activated for degranulation
IL-10	Cytokine	None	None	Participates in and regulates the immune defense response of NK cells during chronic viral and bacterial infections in the body, regulates the dialog between NK cells and dendritic cells at the maternal-fetal interface, and maintains the coordination of the uterine immune environment
IFN-γ	Cytokine	None	None	Promotes innate immunity and adaptive immunity for host protection, is one of the most important cytokines secreted by endometrial NK cells, promotes the maturation of endometrial NK cells, and participates in the remodeling of the uterine spiral arteries in the decidual stage

Ig: immunoglobulin; FcεRIγ: Fc epsilon receptor type Iγ; HLA: human leukocyte antigens; ADCC: antibody-dependent cell-mediated cytotoxicity; KIR: killer cell immunoglobulin-like receptor; SHP: small heterodimer partner; MICA: major histocompatibility complexes class I chain-related molecule A; MICB: major histocompatibility complexes class I chain-related molecule B; ULBP: UL16 binding proteins; DAP: diaminopimelic acid; IL-10: Interleukin 10; IFN-γ: Interferon gamma.

Supplementary table 2. Antibodies for flow cytometry

	manufacturer	Conjugate	Clone	Catalog
CD3	BD	Alexa Fluor 700	UCHT1	56-0038-82
CD16	BD	PerCP/Cyanine5.5	B73.1	565421
CD56	BD	PE-Cy™7	B159	557747
CD27	BD	Brilliant Violet 421	M-T271	562513
CD69	BD	Brilliant Violet 510™	FN50	747521
CD107a	BD	Brilliant Violet 421	H4A3	562623
KIR2DL2/L3/S2	R&D	Alexa Fluor 750	180704	FAB1848S-100UG
KIR2DL4	NOVUS	Alexa Fluor® 647/APC-A	181703	FAB2238A
NKG2C	BD	Alexa Fluor® 647/APC-A	134522	FAB1381R-100UG
NKG2D	BD	PE	1D11	557940
IL-10	BD	Brilliant Violet 650	JES3-9D7	564051
IFN-γ	BD	FITC	4S.B3	502506

Supplementary table 3. Frequencies (%) of cytotoxic NK cell subsets

	pbNK cells			uNK cells		
	$CD56^{dim}CD16^{+}$	$CD56^{bright}CD16^{-}$	$CD56^{dim}CD16^{-}$	$CD56^{bright}CD16^{-}$	$CD56^{bright}CD16^{dim}$	$CD56^{dim}CD16^{bright}$
**ENP**	69.9±13.1	7.7±1.2	5.6±0.5	62.7±14.8	32.6±7.4	3.9±1.4
**EPL**	73.5±15.3	7.0±1.9	6.3±1.5	58.3 ±9.3	34.3±15.0	4.5±2.1
**P value**	0.098	0.104	0.765	0.111	0.412	0.536

EPL: early pregnancy loss group; ENP: early normal pregnancy group. The data are expressed as the mean ± SD, and the data of the two groups were analyzed by the nonparametric Mann–Whitney U test..
